# Translocation t(14;16) in multiple myeloma: gangster or just part of the gang?

**DOI:** 10.1038/s41408-024-00978-z

**Published:** 2024-01-16

**Authors:** Hira Mian, Martin Kaiser, Rafael Fonseca

**Affiliations:** 1 Department of Oncology, McMaster University, Ontario, Canada; 2 Division of Genetics and Epidemiology, The Institute of Cancer Research, London, UK; 3 Department of Haematology, The Royal Marsden Hospital, London, UK; 4 Division of Hematology and Medical Oncology, Mayo Clinic in Arizona, Phoenix, Arizona USA

**Keywords:** Genetics research, Translational research

Dear Editor,

It is with great interest that we read the article by Schavgoulidze et al. proposing a lack of prognostic significance of the translocation t(14;16) amongst newly-diagnosed multiple myeloma (MM) [1]. As previously reported by others, they show t(14;16) was commonly present with other progression genetic events such as deletion 17p, gain/amp 1q and deletion 1p32. The authors conclude that only t(14;16) associated with other concurrent genetic progression abnormalities should be considered high-risk. Here we argue that t(14;16): (1) should continue to be considered high-risk regardless, and (2) continue to be routinely included in test profiles that identify high-risk subgroups.

MM genetic abnormalities are considered to be either primary (immunoglobulin heavy chain (IgH) translocations and trisomies leading to hyperdiploidy) or secondary (monosomies/deletion and amplifications). Primary translocations are present in all cells and endure for the duration of the disease. They are thought to be an early initiating event in transformation of a pre-malignant to a malignant plasma cell clone [2]. Partner loci to these IgH translocations include 11q13 (CCND1), 6p21 (CCND3), 4p16 (FGFR3/NSD2), 16q23 (c-MAF), and 20q12 (MAFB) [3]. These translocations lead to juxtaposition of the strong IgH enhancer with these MM oncogenes, driving their pathologic overexpression. Specifically in t(14;16), the IgH enhancer drives overexpression of the oncogenic transcription factor c-musculoaponeurotic fibrosarcoma (c-MAF) and hard-wires a transcriptional program into every single cell of the tumor, for all subsequent cell divisions to come [4]. Not surprisingly, consistently across generations of myeloma researchers, and using more refined genetic technologies, the same truth emerges: biological sub-groups of MM divide primarily along these early events, IgH translocations and hyperdiploidy.

The perilous, downstream, genetic consequences of the t(14;16) are impressive and have been well described. The most striking recent finding is the characteristic APOBEC mutational signature with increased expression of APOBEC3A and APOBEC3B [5]. These enzymes, while protective against pathogens by mutating their genomes, are thought to contribute towards cancer genomic instability, resulting in more diverse and adaptable/aggressive MM genomes. The MM genomic instability conferred by APOBEC stick to t(14;16) like a piece of DNA evidence to the crime scene, including a high rate of secondary genetic lesions and mutational burden. Not only does the t(14;16) carry the highest frequency of triple and quadruple hits at diagnosis, but in longitudinal studies of matched samples this subgroup acquires the most additional secondary genetic changes [6]. This is in stark contrast with the t(11;14) tumors which see the lowest proportion of acquired changes at relapse.

Inherently, all studies on the clinical impact of t(14;16) are limited by their low frequency (<5%). Analyses are further hampered by the rapid evolution of MM treatments. Amongst these we now see routine use of combination therapies, and lenalidomide maintenance. The dramatic effects of specific genetic sub-groups on MM survival have only recently, and with adequate long-term follow-up [7], become visible. Large clinical trials with controlled, highly homogeneous treatment are required to reliably elucidate prognostic associations, especially of very rare tumor sub-groups. It is in such trials that the changing prognostic impact of adverse lesions in isolation (single hit) in context of effective therapy such as lenalidomide maintenance or combination consolidation therapy, has consistently been demonstrated [7–9]. However, these studies have not diminished the value of testing for and recognizing t(14;16) as a fundamentally adverse player at all. In the cohort described by Schavgoulidze et al., there was heterogeneity in treatment regimens received by patients including both transplant eligible and ineligible regimen combinations. Additionally, the duration of lenalidomide maintenance is not known. This is consistent with other studies where a more modest impact may be observed with isolated/single hit genetic events with different therapeutic modalities [7, 10, 11] and particularly for infrequent markers such as t(14;16). Similarly, in the second revision of the international staging system (R2-ISS) which utilized 15 pooled clinical trials for model building, while t(14;16) showed a trend towards a shorter PFS, it was not statistically significant (HR 1.15 [95% CI, 0.96 to 1.37], *P* = 0.13) which could have potentially been impacted by the heterogeneity of the dataset and the therapies received [12].

The targeting of primary genetic lesions remains one of our best opportunities for developing biology driven interventions - potential preventive (secondary) or curative. As shown in the case of t(11;14) and BCL-2 inhibitors [13], tailored therapeutic strategies need to be continually developed and evaluated in MM. Not profiling the distinct biological and clinical effects of t(14;16) may delay targeted therapeutic approaches for this subgroup.

Furthermore, the impact of isolated genetic events on treatment decisions such as escalation and de-escalation remains unknown. For example, while a modest impact of t(14;16) may be seen in isolation among patient treated with quadruplet therapy and continuous maintenance, a much more detrimental impact may be noted in this same cohort with de-escalation of maintenance therapy or limited duration therapy. As we now have the luxury of evaluating de-escalation strategies in MM given the advances made in therapeutics, it is particularly important to identify distinct biological and clinical subgroup of patients such as those with t(14;16) who may be uniquely impacted by these treatment strategies.

Within every genetic subgroup of MM one is bound to find prognostic outliers—bad outcomes among those with putative good genetic risk profiles, and good outcomes even among patients with high-risk genetics. However, the more common associations and outcome should prevail. For the t(14;16) the preponderance of evidence suggest it is a high-risk markers, and one of the ones with the greatest impact on outcomes.

In summary, we affirm our belief that the t(14;16), as a primary translocation event, should remain an indicator of high-risk disease. It is not simply a by-stander ‘gang member’ amidst the genetic chaos but rather the instigator classifying it as a ‘gangster’. We also reiterate that the t(14;16) should continue to be routinely profiled.

## References

[1] Schavgoulidze A, Perrot A, Cazaubiel T, Leleu X, Montes L, Jacquet C (2023). Prognostic impact of translocation t(14;16) in multiple myeloma according to the presence of additional genetic lesions. Blood Cancer J.

[2] Fonseca R, Blood E, Rue M, Harrington D, Oken MM, Kyle RA (2003). Clinical and biologic implications of recurrent genomic aberrations in myeloma. Blood.

[3] Kumar SK, Rajkumar SV (2018). The multiple myelomas - current concepts in cytogenetic classification and therapy. Nat Rev Clin Oncol.

[4] Hurt EM, Wiestner A, Rosenwald A, Shaffer AL, Campo E, Grogan T (2004). Overexpression of c-maf is a frequent oncogenic event in multiple myeloma that promotes proliferation and pathological interactions with bone marrow stroma. Cancer Cell.

[5] Walker BA, Wardell CP, Murison A, Boyle EM, Begum DB, Dahir NM (2015). APOBEC family mutational signatures are associated with poor prognosis translocations in multiple myeloma. Nat Commun.

[6] Croft J, Ellis S, Sherborne AL, Sharp K, Price A, Jenner MW (2021). Copy number evolution and its relationship with patient outcome—an analysis of 178 matched presentation-relapse tumor pairs from the Myeloma XI trial. Leukemia.

[7] Panopoulou A, Easdale S, Ethell M, Nicholson E, Potter M, Giotas A (2023). Impact of ultra high-risk genetics on real-world outcomes of transplant-eligible multiple myeloma patients. Hemasphere.

[8] Benboubker L, Dimopoulos MA, Dispenzieri A, Catalano J, Belch AR, Cavo M (2014). Lenalidomide and dexamethasone in transplant-ineligible patients with myeloma. N. Engl J Med.

[9] McCarthy PL, Holstein SA, Petrucci MT, Richardson PG, Hulin C, Tosi P (2017). Lenalidomide maintenance after autologous stem-cell transplantation in newly diagnosed multiple myeloma: a meta-analysis. J Clin Oncol.

[10] Costa LJ, Chhabra S, Medvedova E, Dholaria BR, Schmidt TM, Godby KN (2021). Daratumumab, Carfilzomib, Lenalidomide, and Dexamethasone with minimal residual disease response-adapted therapy in newly diagnosed multiple myeloma. J Clin Oncol.

[11] Mina R, Musto P, Rota-Scalabrini D, Paris L, Gamberi B, Palmas A (2023). Carfilzomib induction, consolidation, and maintenance with or without autologous stem-cell transplantation in patients with newly diagnosed multiple myeloma: pre-planned cytogenetic subgroup analysis of the randomised, phase 2 FORTE trial. Lancet Oncol.

[12] D’Agostino M, Cairns DA, Lahuerta JJ, Wester R, Bertsch U, Waage A (2022). Second revision of the International Staging System (R2-ISS) for overall survival in multiple myeloma: a European Myeloma Network (EMN) report within the Harmony project. J Clin Oncol.

[13] Kumar SK, Harrison SJ, Cavo M, de la Rubia J, Popat R, Gasparetto C (2020). Venetoclax or placebo in combination with bortezomib and dexamethasone in patients with relapsed or refractory multiple myeloma (BELLINI): a randomised, double-blind, multicentre, phase 3 trial. Lancet Oncol.

